# Time Trends in Epidemiologic Characteristics and Imaging Features of Lung Adenocarcinoma: A Population Study of 21,113 Cases in China

**DOI:** 10.1371/journal.pone.0136727

**Published:** 2015-08-28

**Authors:** Li Zhang, Meng Li, Ning Wu, Yuheng Chen

**Affiliations:** 1 Department of Diagnostic Radiology, Cancer Hospital, Chinese Academy of Medical Sciences, Peking Union Medical College, Beijing, China; 2 PET-CT Center, Cancer Hospital, Chinese Academy of Medical Sciences, Peking Union Medical College, Beijing, China; 3 Cancer Foundation of China, Cancer Hospital, Chinese Academy of Medical Sciences, Peking Union Medical College, Beijing, China; Peking University People Hospital, CHINA

## Abstract

**Objectives:**

This study aims to describe time trends of epidemiologic characteristics and imaging features over 14 years among histologically confirmed lung adenocarcinoma (ADC) in China and to discuss the possible reasons for these changes.

**Materials and Methods:**

Data of 21,113 pathologically confirmed lung cancer patients from January 1999 to December 2012 were analyzed retrospectively. Preoperative high-resolution computer tomography (HRCT) images were available and reviewed in 5,439 lung ADC patients since 2005. Time trends of the ADC proportion of lung cancer cases, gender distribution, age at diagnosis, the proportion of early-stage ADC and imaging features were investigated.

**Results:**

The proportion of ADC increased during the 14 years (*P* = 0.000). The ratio of female to male ADC cases was higher than both squamous cell carcinoma (SQCC) and total lung cancer cases (*P* = 0.000). The median age at diagnosis of ADC patients was younger than that of both SQCC and total lung cancer during the 14 years (*P* = 0.000). The proportion of age group 45–59 years increased in total lung cancer cases (*P* = 0.000). When stratified by lung cancer histopathologic subtypes, this trend was also observed in ADC (*P* = 0.001) and SQCC (*P* = 0.007). The proportion of early-stage cases of ADC increased from 2008 to 2012 (*P* < 0.001). The proportion of subsolid nodules (SSN) in ADC increased (*P* = 0.001) from 2005 to 2012.

**Conclusion:**

The data suggests that the proportion of ADC increased from 1999 to 2012 especially in middle-aged, female patients; early-stage ADC and SSN on HRCT images gradually increased, which may have been caused by a change in smoking habits and increased application of HRCT.

## Introduction

Lung cancer is the most commonly diagnosed cancer and the most common cause of cancer death throughout the world [1]. In China, lung cancer has replaced liver cancer as the first cancer-related cause of death among patients with malignant tumors [2]. According to the Chinese Cancer Registry Annual Report 2012 [3], approximately 640,000 people die of lung cancer each year in China. Among them, adenocarcinoma (ADC) is the most frequent histopathologic type, which accounts for almost half of the diagnosed cases [4]. In lung cancer CT baseline screening, the proportion of ADC among all cancer diagnoses reaches as high as 76%[5].

Trends in the histopathology of lung cancer have been observed in various countries [6–9], including an upward trend in ADC incidence. However, there has been no further research on time trends of ADC in relation to epidemiologic characteristics and imaging features. This paper describes the time trends of ADC over a 14-year period among 21,113 cases of ADC at the Cancer Hospital of Chinese Academy of Medical Sciences (CHCAMS) and discusses the underlying reasons for the observed changes. It is hoped that this investigation can enable better understanding of ADC and establish further ADC research.

## Materials and Methods

### Study subjects and data collection

Our institutional ethics committee approved this retrospective study and determined that no informed consent was required due to the retrospective nature of the study and the fact that the data were going to be analyzed anonymously.

Data from 21,113 consecutive lung cancer patients in CHCAMS between January 1999 and December 2012 were retrospectively reviewed. All patients were pathologically confirmed to have lung cancer, in which 12,241 cases underwent surgical resection and 8,872 cases underwent small biopsies and/or cytology. The demographic epidemiologic data, including gender and age at diagnosis, as well as clinical characteristics, such as date of operation and pathological results, were collected from CHCAMS.

### Epidemiologic characteristics

According to the WHO Classification of Tumors 2004 [10], the cases were categorized into nine histological subtypes: ADC, squamous cell carcinoma (SQCC), small cell lung cancer (SCLC), adenosquamous carcinoma (ADSQC), large cell carcinoma (LCC), sarcomatoid carcinoma (SAC), carcinoid tumor (CCT), salivary gland tumors (SGTs) and preinvasive lesions (PIL). For tumors that did not meet any criteria of the above nine subtypes, the terminology “lung cancer-not otherwise specified” (LC-NOS) was used. To assess the changes in the trends of the proportion of ADC, we combined SCLC, ADSQC, LCC, SAC, CCT, SGTs, PIL and LC-NOS as one group of other subtypes and compared the ADC cases to this group or SQCC. The ADC and SQCC proportion in both male and female patients was compared. Gender distribution and changing trends in ADC, SQCC, and total lung cancer cases from 1999 to 2012 were analyzed and compared. The ratio of female to male patients and age at diagnosis in ADC, SQCC and the total lung cancer cases during the 14-year period were investigated and compared. According to the WHO age classification 2004 [11], study subjects were divided into three age groups: young people (≤ 44 years), middle-aged people (45–59 years) and elderly people (≥ 60 years). The difference among these three groups was analyzed.

### The proportion of early-stage ADC cases

In our study, early-stage ADC cases were defined as pathological T_1_N_0_M_0_ (p T_1_N_0_M_0_) according to the seventh edition of TNM Classification of Malignant Tumors published by the International Union Against Cancer (UICC) and the American Joint Committee on Cancer (AJCC). The proportion of early-stage ADC cases was calculated each year, and the time trends during 1999 to 2012 analyzed.

### Imaging features

Pre-treatment HRCT images in the picture archiving and communication systems (PACS) of ADC were reviewed. Because PACS in CHCAMS were established in 2005, we only included the preserved images from 2005 to 2012. These cases were divided into two groups according to imaging features: solid nodules (SN) and subsolid nodules (SSN). SN was defined as those that completely obscure the lung parenchyma. SSN, including pure ground-glass nodules (GGN) and part-solid nodules (PSN), were defined as focal nodular areas of increased lung attenuation, through which or part of which normal parenchymal structures such as airways, vessels, and interlobular septa can be defined [12, 13]. The SSN proportion was calculated each year, and the time trends throughout the time period 2005 to 2012 were analyzed.

### Statistical methods

The differences in proportion of histopathology subtypes, gender, age groups, proportion of early-stage cases and imaging features were evaluated by Chi-Square test. The difference in age distribution among 14 years was analyzed using Wilcoxon Rank Sum test. The association between the proportion and year period was analyzed using simple linear regression analysis. SPSS statistical software version 17.0 (SPSS, Inc., an IBM Company, Chicago, IL, USA) was used for all data analyses. Differences were considered significant when two-sided *P*-values were less than 0.05.

## Results

### Time trends in histological types of lung cancer

**Fig 1:**
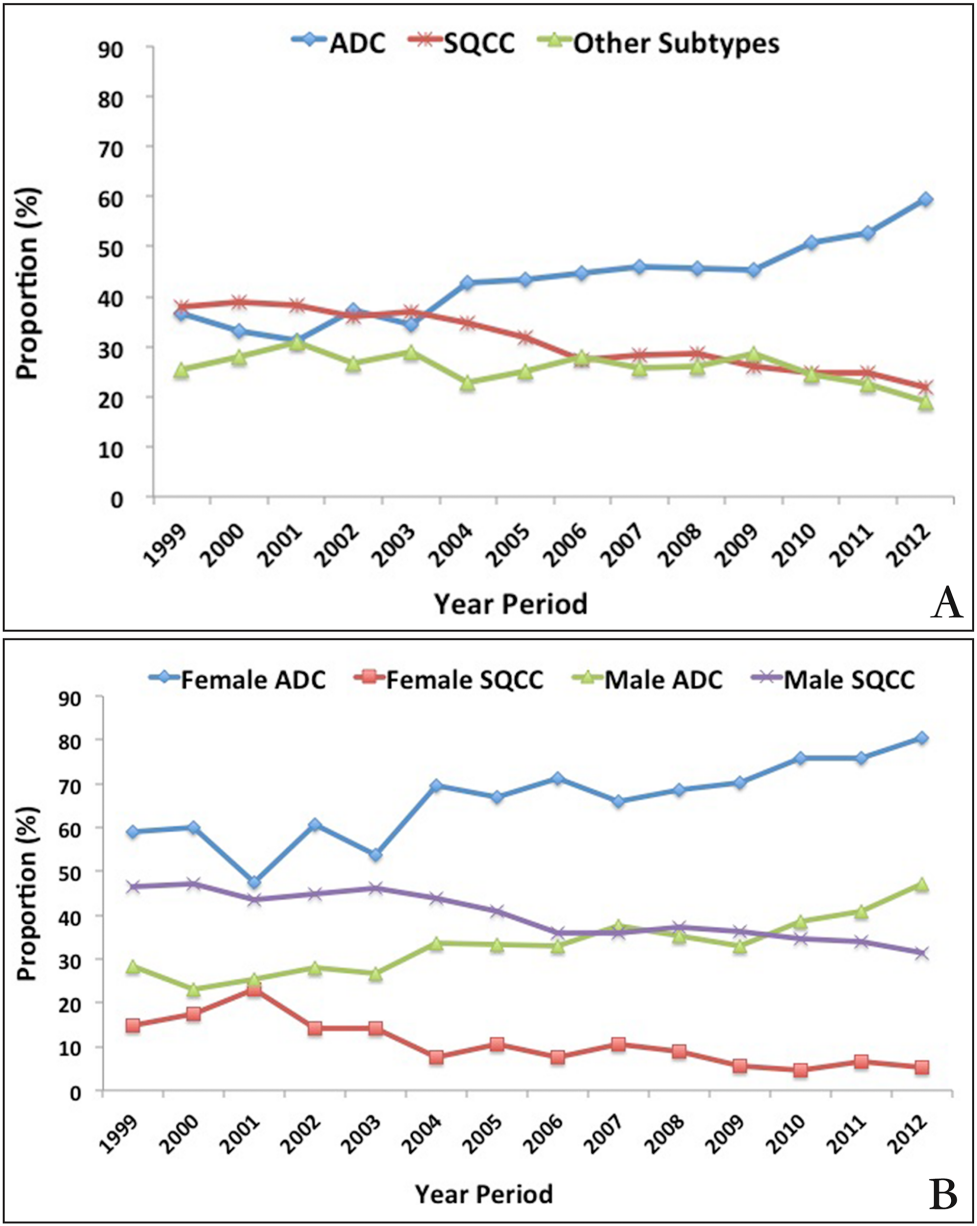
A. The proportion of ADC, SQCC and other subtypes in CHCAMS from 1999 to 2012; B. The proportion of ADC, SQCC stratified by females and males in CHCAMS from 1999 to 2012.

A total of 21,113 pathological confirmed lung cancer cases were recorded in the study (Table 1). As expected, the number of patients with lung cancer increased year by year. Subtypes of lung cancer among 14 years were significantly different (X^2^ = 1023.31, *P* = 0.000). ADC and SQCC were the main subtypes, which accounted for more than 70% of all the cases. Between 1999 and 2012, the proportion of ADC increased 22.6% (from 36.7% to 59.3%) and SQCC decreased 16.1% (from 37.8% to 21.7%), while other subtypes remained stable (Fig 1A). A simple linear correlation between ADC proportion and year period was observed with a formula Ŷ (ADC proportion) = 1.77X (Year Period)– 3509.99 (R^2^ = 0.87, F = 79.13, *P* = 0.000). The proportion of ADC increased yearly in both females and males (Fig 1B). In female patients, the proportion of ADC was remarkable higher than that of SQCC since 1999. In male patients, ADC was lower than SQCC until 2009 and thereafter higher than SQCC.

**Table 1:** Distribution of histology among pathologically confirmed lung cancer cases in CHCAMS from 1999–2012.

Year	ADC (n, %)	SQCC (n, %)	SCLC (n, %)	ADSQC (n, %)	LCC (n, %)	SAC (n, %)	CCT (n, %)	SGTs (n, %)	PIL (n, %)	LC-NOS (n, %)
1999	263 (36.7)	271 (37.8)	89 (12.4)	46 (6.4)	4 (0.6)	0 (0.0)	8 (1.1)	3 (0.4)	0 (0.0)	32 (4.5)
2000	262 (33.2)	306 (38.8)	126 (16.0)	30 (3.8)	9 (1.1)	0 (0.0)	10 (1.3)	9 (1.1)	0 (0.0)	36 (4.6)
2001	283 (31.2)	345 (38.1)	145 (16.0)	56 (6.2)	13 (1.4)	3 (0.3)	9 (1.0)	5 (0.6)	0 (0.0)	47 (5.2)
2002	336 (37.3)	325 (36.1)	116 (12.9)	52 (5.8)	9 (1.0)	1 (0.1)	6 (0.7)	5 (0.6)	1 (0.1)	50 (5.5)
2003	305 (34.4)	326 (36.8)	126 (14.2)	53 (6.0)	9 (1.0)	3 (0.3)	6 (0.7)	4 (0.5)	0 (0.0)	54 (6.1)
2004	417 (42.6)	340 (34.7)	105 (10.7)	35 (3.6)	20 (2.0)	4 (0.4)	4 (0.4)	1 (0.1)	1 (0.1)	52 (5.3)
2005	586 (43.3)	430 (31.8)	196 (14.5)	27 (2.0)	8 (0.6)	2 (0.1)	5 (0.4)	3 (0.2)	0 (0.0)	97 (7.2)
2006	651 (44.5)	401 (27.4)	240 (16.4)	19 (1.3)	16 (1.1)	4 (0.3)	8 (0.5)	5 (0.3)	2 (0.1)	118 (8.1)
2007	773 (46.1)	472 (28.2)	250 (14.9)	29 (1.7)	17 (1.0)	4 (0.2)	7 (0.4)	7 (0.4)	0 (0.0)	117 (7.0)
2008	801 (45.5)	504 (28.6)	269 (15.3)	28 (1.6)	20 (1.1)	9 (0.5)	12 (0.7)	6 (0.3)	1 (0.1)	111 (6.3)
2009	909 (45.2)	525 (26.1)	311 (15.5)	25 (1.2)	12 (0.6)	11 (0.5)	10 (0.5)	9 (0.4)	4 (0.2)	194 (9.7)
2010	1192 (50.7)	583 (24.8)	323 (13.7)	40 (1.7)	13 (0.6)	13 (0.6)	14 (0.6)	3 (0.1)	1 (0.0)	169 (7.2)
2011	1396 (52.8)	652 (24.7)	391 (14.8)	23 (0.9)	24 (0.9)	18 (0.7)	13 (0.5)	9 (0.3)	3 (0.1)	114 (4.3)
2012	1588 (59.3)	582 (21.7)	285 (10.6)	27 (1.0)	27 (1.0)	24 (0.9)	19 (0.7)	11 (0.4)	3 (0.1)	112 (4.2)
Total	9762 (46.2)	6062 (28.7)	2972 (14.1)	490 (2.3)	201 (1.0)	96 (0.5)	131 (0.6)	80 (0.4)	16 (0.1)	1303 (6.2)

ADC, adenocarcinoma; SQCC, squamous cell carcinoma; SCLC, small cell lung cancer; ADSQC, adenosquamous carcinoma; LCC, large cell carcinoma; SAC, sarcomatoid carcinoma; CCT, carcinoid tumor; SGTs, salivary gland tumors; PIL, preinvasive lesions; LC-NOS, lung cancer-not otherwise specified.

### Time trends in gender distribution of ADC

**Fig 2:**
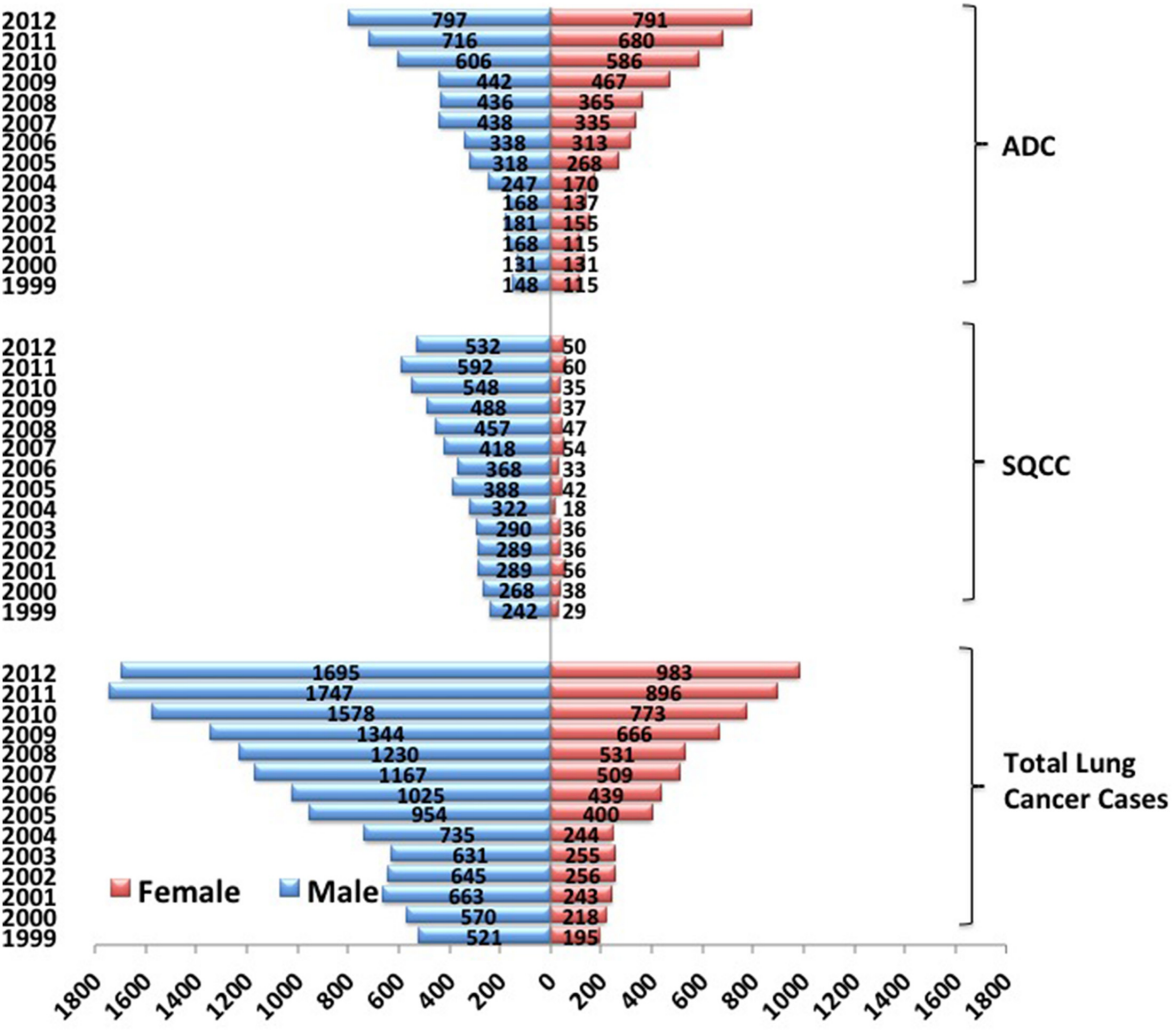
The gender distribution of patients with ADC or SQCC or of the total lung cancer patients in CHCAMS from 1999 to 2012.

There were 5,134 male (52.60%) and 4,628 female (47.40%) cases in the total 9,762 ADC cases. The gender distribution among the 14 years was significantly different (X^2^ = 34.53, *P* = 0.001). The ratio of female to male with ADC was 0.90, which was significantly higher than that of both SQCC and total lung cancer cases (SQCC: ratio = 0.10, X^2^ = 2446.46, *P* = 0.000; total lung cancer cases: ratio = 0.46, X^2^ = 748.44, *P* = 0.000) (Fig 2). A positive, but not significant, correlation between the ratio of female to male patients with ADC and year period was observed during the 14 years (R = 0.51, *P* = 0.06). However, a negative correlation and a positive correlation were found between the ratio and year period with SQCC and total lung cancer cases, respectively (SQCC: R = -0.60, *P* = 0.02; total lung cancer cases: R = 0.88, *P* = 0.000).

### Time trends in age at diagnosis of ADC

The median age at diagnosis of the 9,762 ADC patients was 58 years with a range from 11 to 90 years, which was younger than that of both SQCC (median: 61, range: 25–92; Z = -14.80, *P* = 0.000) and the total lung cancer cases (median: 59, range: 11–92; Z = -4.93, *P* = 0.000). No statistically significant differences in age at diagnosis of ADC patients were observed between 1999 and 2012 (X^2^ = 16.78, *P* = 0.21). Although the age group of ≥ 60 years was the largest group in all three groups (ADC, SQCC and total lung cancer cases), the age group constituent in ADC was significantly different from that in both SQCC and total lung cancer cases (ADC *vs*. SQCC: X^2^ = 237.99, *P* = 0.000; ADC *vs*. total lung cancer cases: X^2^ = 20.91, *P* = 0.000). The proportion of patients of age ≤ 44 years decreased during the 14-year period among the ADC, SQCC and total lung cancer cases, while patients of 45–59 years increased and those ≥ 60 years remained stable. A positive correlation between the proportion of patients of 45–59 years and the 14-year period was observed for ADC, SQCC and total lung cancer cases, while a negative correlation between the proportion of patients ≤ 44 years and the 14-year period was also observed (Table 2).

**Table 2:** Correlation between age group proportion and year period of lung cancer patients in CHCAMS from 1999–2012.

	≤ 44 years		45–59 years		≥ 60 years	
	*r*	*P*	*r*	*P*	*R*	*P*
ADC	-0.68	0.008	0.77	0.001	-0.35	0.220
SQCC	-0.88	0.000	0.68	0.007	-0.15	0.600
Total Lung Cancer Cases	-0.67	0.009	0.87	0.000	-0.54	0.046

### Time trends in the proportion of early-stage cases

**Fig 3:**
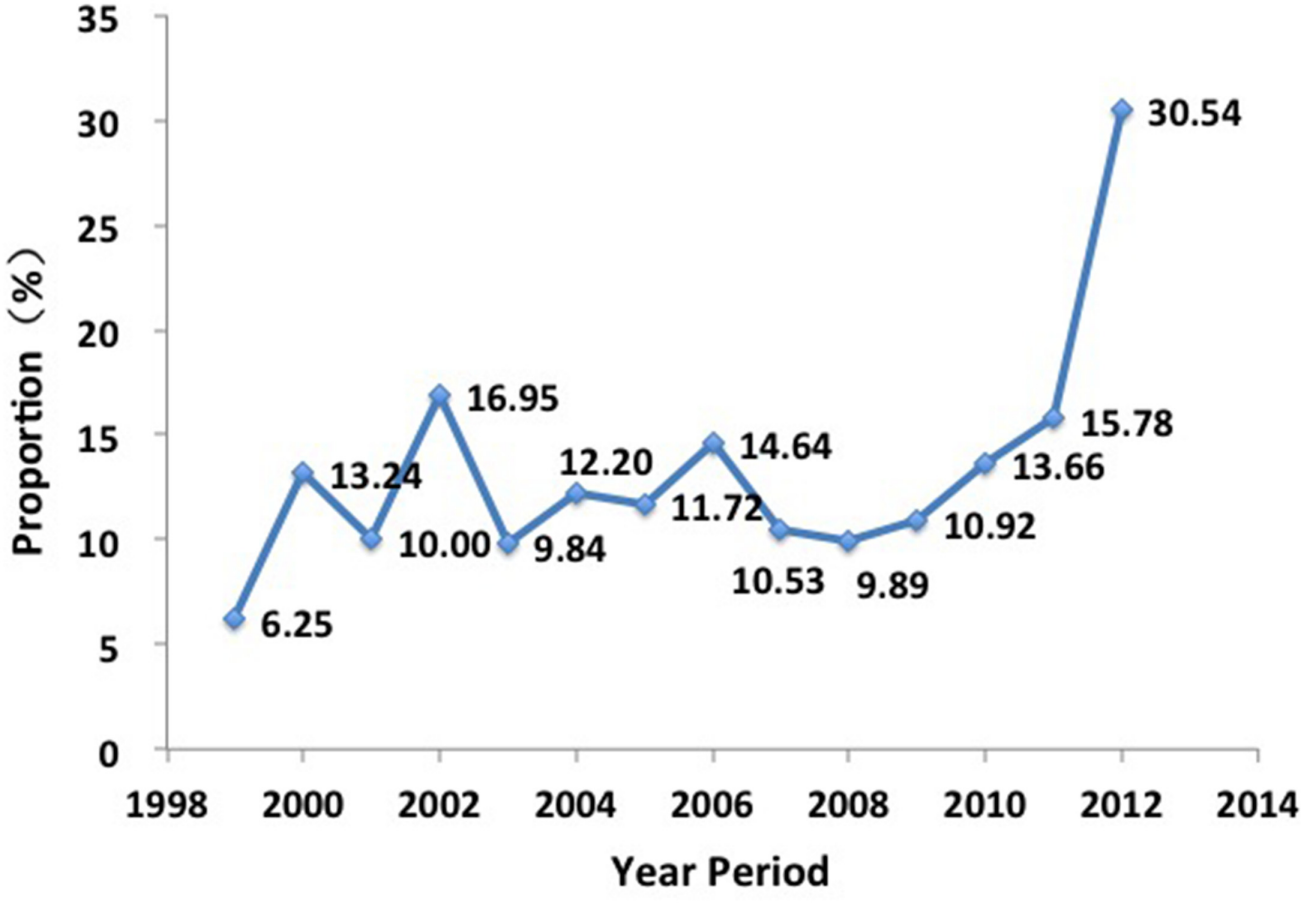
Distribution of early-stage ADC in CHCAMS from 1999 to 2012.

7,202 of 9,762 ADC patients had pathological TNM staging, in which 16.0% (1,155 of 7,202) were early-stage cases. Significant differences in the proportion of early-stage cases were observed among14 years (X^2^ = 279.762, *P* < 0.001). A positive correlation (*r* = 0.9, *P* < 0.001) between the proportion of early-stage cases and year period was observed during 2008 to 2012 (Fig 3).

### Time trends in imaging features of ADC

**Fig 4:**
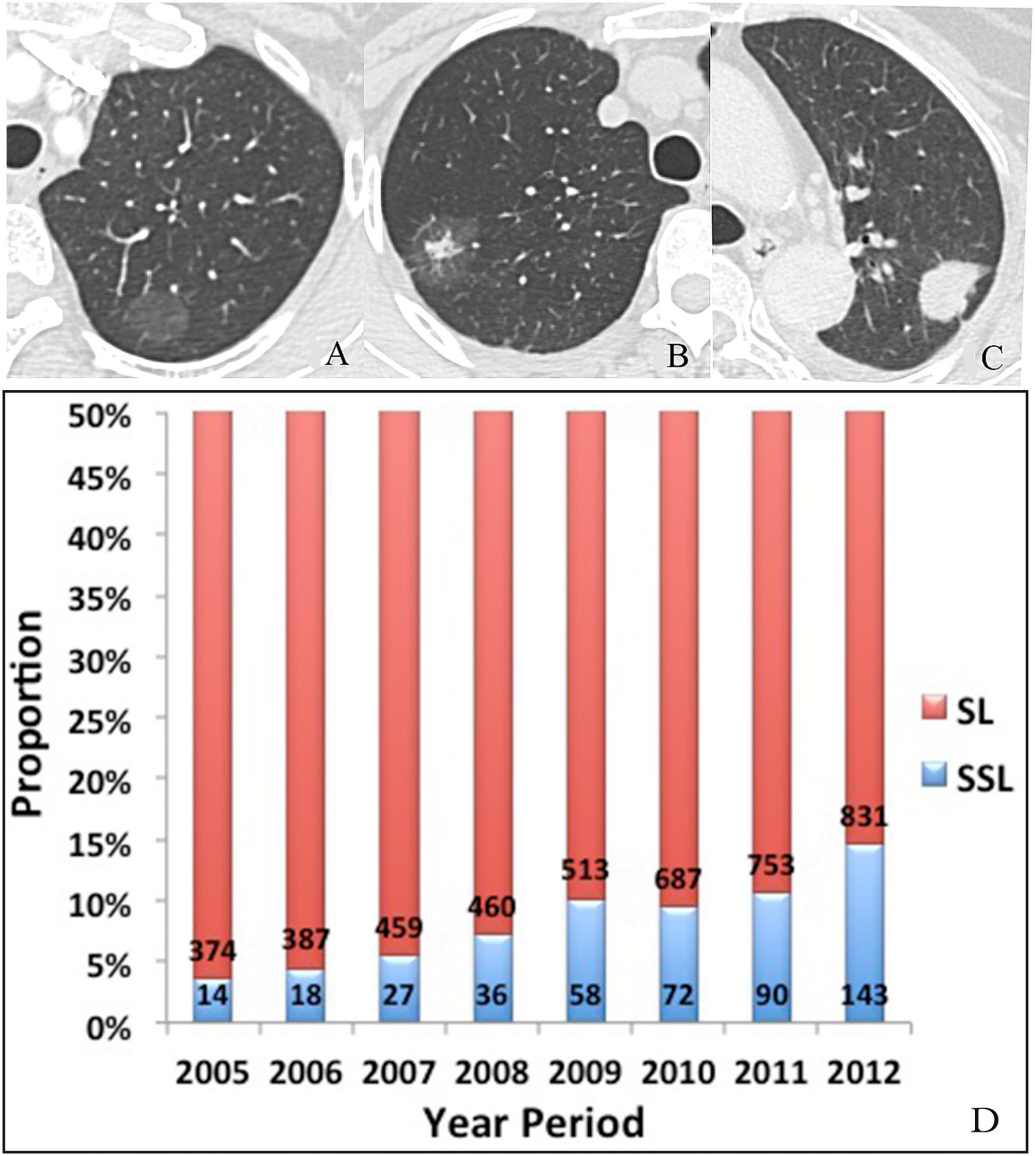
A-C: Various imaging features of ADC in HRCT images, D: Distribution of ADC with SN and SSN in CHCAMS from 2005 to 2012. A. pure ground-glass nodule, B. part-solid lesion, C. solid nodule.

The pre-treatment HRCT images for 5,439 of 9,762 ADC patients from 2005 to 2012 were reviewed. According to the imaging features, 483 cases were SSN and 4,956 cases were SN. The proportion of SSN increased during the period from 2005 to 2012 (Fig 4). A simple linear correlation between the proportion and year period was observed with a formula Ŷ (SSN proportion) = 1.27X (Year Period)– 2538.80 (R^2^ = 0.87, F = 40.13, *P* = 0.001).

## Discussion

Using data from CHCAMS, we investigated epidemiologic characteristics and imaging features trends in lung ADC over a 14-year period. In this study, all patients had pathologically confirmed lung cancer, which ensure the reliability of the data. In addition, CHCAMS is a supporting institution of the National Cancer Center of China and the largest center of cancer prevention and treatment in Asia. Patients in this hospital came from all over China. Moreover, the percent distribution by histology in our study is consistent with Surveillance, Epidemiology, and End Results Program (SEER)[14] and a meta-analysis of lung cancer in China based on published data from 1990 to 2011 [15]. From this point of view, our data can represent some aspects of the lung cancer status of China.

We observed that the proportion of ADC increased since 1999 while SQCC decreased in both men and women. The proportion of ADC has surpassed SQCC and become the largest proportion of lung cancer since 2009 in our study. These results are consistent with those observed based on the data from Shenyang in China [6], the Midwestern United States [7], Osaka in Japan [8], and Canada [9]. The increase in ADC proportion could be attributed to the aspects below. First, ADC usually develops in the peripheral parenchyma of the lung [16]. The improved diagnostic ability of peripheral tumors, by CT and transthoracic aspiration needle biopsy, may increase the number of ADC cases diagnosed [9, 17, 18]. Second, filtered cigarettes have gradually replaced most non-filtered cigarettes since 1980. Filtered cigarettes with less tar and nicotine are inhaled more deeply than non-filtered cigarettes, which may allow the smoke and carcinogens to infiltrate the peripheral lung tissues where the majority of ADCs normally arise [19]. Finally, the composition of modern cigarette smoke has changed [20]. The high level of polynuclear aromatic hydrocarbons (PAH) in the smoke of the modern cigarettes has been replaced by organ specific carcinogenic tobacco specific nitrosamines (TSNAs). The metabolite of TSNA, 4-methylnitrosamino-1-3-pyridil-1-butanone (NNK), may induce the formation of ADC [21, 22], while PAH may induce SQCC in animal experiments [23]. Reported risk factors [24–33], including passive cigarette smoking; exposure to cooking fumes, air pollution, asbestos, and radon; nutritional status; genetic susceptibility; immunologic dysfunction; tuberculosis infection; asthma; and human papillomavirus infection may play roles in the observed increase in lung ADC.

In our study, the ratio of female to male patients in all lung cancer cases increased significantly since 1999. In ADC cases, a positive but not significant correlation between this ratio and year period was observed. This may be due to a large number of non-smoking females that are exposed to tobacco in China, compared with males. Based on our unpublished data, among 6,203 lung cancer screening subjects with complete information on smoking, the passive smoking risk of females was higher than males (odds ratio: 2.83, 95% confidence interval: 2.68–3.00). The smoking exposure risk (including active smoking and passive smoking) of females compared with males increased from 2007 to 2013. Yang *et al*.[34] also reported that the proportion of passive smoking in females was higher than that in males and, moreover, women are more susceptible to tobacco-related lung cancer compared to men, according to Henschke’s report [35]. Other risk factors, including cooking fumes, indoor air pollutants and family history of lung cancer, may contribute to female lung ADC [36, 37].

To the best of our knowledge, we observed, for the first time, that patients with ADC are younger than patients with either SQCC or total lung cancer cases. Further studies are warranted to elucidate the reasons for this phenomenon. The proportion of age group of 45–59 years with ADC increased from 1999 to 2012 and a positive correlation between the proportion and year period was observed not only in ADC, but also in SQCC and the total lung cancer cases. This observation may be related to the increased detection of early-stage lung cancer.

In our study, the proportion of early-stage cases increased from 9.89% in year 2008 to 30.54% in year 2012. The main contributor of this increase may be that more and more people are aware of the necessity of cancer screening. Meanwhile, the increased availability of HRCT and the advent of low-dose CT screening programs has improved early detection of lung cancer [38]. In our hospital, the number of subjects with lung cancer screening increased from 141 in year 2007 to 2,170 in year 2012. Church *et al*.[39]reported that the proportion of early-stage lung cancer in detected lung cancer cases was higher in the low-dose CT screening group (158 of 292 [54.11%]) than in the radiography screening group (70 of 190 [36.84%]), which confirmed the superiority of low-dose CT screening in detecting early-stage lung cancer cases [40, 41].

Early-stage ADC may present as SSN on HRCT images. In our study, the proportion of SSN increased during the period from 2005 to 2012, and a simple linear correlation between the proportion and year period was observed. In the year of 2012, the proportion of SSN reached as high as 14% (143 of 974). According to the new International Multidisciplinary Classification of Lung ADC [42], SSN on HRCT images can represent different kinds of ADC subtypes, including atypical adenomatous hyperplasia, adenocarcinoma in situ, minimally invasive adenocarcinoma and some invasive adenocarcinomas with a predominant lepidic pattern. However, studies [43–45] have shown that different ADC subtypes exhibit different clinical prognoses that may lead to different surgical procedures and adjuvant treatment strategies. Advanced research is necessary to develop reasonable individual treatment plans for SSN.

In summary, our study shows that the proportion of ADC increased from 1999 to 2012 especially in middle-aged, female patients. In addition, early-stage lung ADC and SSN on CT images gradually increased. According to these trends, female patients and the age group of 45–59 years might need to be paid more attention to in lung cancer screening. Further studies are warranted to understand the exact reasons for the increase in lung ADC and the implementation of a specific intervention may be needed. Because of the increase in the proportion of early-stage cases and SSN in ADC, further studies on standardized protocols of management of early-stage cases and SSN are needed.

Our study has several acknowledged limitations. First, this is a single institute study; the data were collected from only one hospital, which cannot represent the lung cancer status for all of China. Further studies using data collected from multiple centers with a longer study period are needed to confirm our results. Second, the risk factors causing the tendency of ADC were not analyzed due to the retrospective nature of the study. A prospective study is warranted to elucidate the exact reasons for the increase in the proportion of ADC.

## Supporting Information

S1 Data Relevant data underlying the findings of time trends in histological types of lung cancer, gender distribution of ADC and age at diagnosis of ADC described in manuscript. (XLS)

S2 Data Relevant data underlying the findings of time trends in the proportion of early-stage cases described in manuscript. (XLS)

S3 Data Relevant data underlying the findings of time trends in imaging features of ADC described in manuscript. (XLS)
